# Integrating Local Knowledge into a National Programme: Evidence from a Community-Based Diabetes Prevention Education Programme

**DOI:** 10.3390/healthcare7010038

**Published:** 2019-03-07

**Authors:** Michele S.Y. Kok, Lisa Bryant, Clare Cook, Sara Blackmore, Mat Jones

**Affiliations:** 1 Centre for Public Health and Wellbeing, University of the West of England, Bristol BS16 1QY, UK; matthew.jones@uwe.ac.uk; 2 South Gloucestershire Council, Bristol BS37 5AF, UK; lisa.bryant@southglos.gov.uk (L.B.); clare.cook@southglos.gov.uk (C.C.); sara.blackmore@southglos.gov.uk (S.B.)

**Keywords:** diabetes prevention, diabetes education, community programme, knowledge integration

## Abstract

Type 2 diabetes prevention is a major priority for healthcare services and public health. This study aimed to evaluate how a local authority in England piloted a diabetes prevention programme. The South Gloucestershire Diabetes Prevention (Pilot) Programme (SGDPP) comprised a group health education course over six weeks with subsequent support provision up to six months post-enrolment. Of the 300 patients invited onto the programme, 32% enrolled and 29% completed the full six-month programme. There was an attendance rate of 84% throughout group sessions and at a six-month follow-up. There were significant improvements across most measures at six months, including a 4 kg mean weight loss and a 3.45 mmol/mol mean HbA1c reduction. Clear goals, high quality organization and personal qualities of educators were identified as central for the programme’s success. The unit costs were similar to pilots of other healthy lifestyle programmes. The evaluation found evidence of reduced type 2 diabetes risk markers, positive impacts for dietary and physical activity, and potential cost-effectiveness for this format of group-based diabetes prevention intervention. Feedback from multiple stakeholders provided insight on how to successfully embed and scale-up delivery of diabetes prevention work. This evidence enables the integration of learning in local service delivery and provides a basis to support development of the national diabetes prevention programme.

## 1. Introduction

Since 1996, the number of people diagnosed with diabetes in the UK has more than doubled from 1.4 million: in 2018, there were 3.8 million people living in the UK diagnosed with diabetes and a further estimated 1 million people living with diabetes, but as yet remain undiagnosed [1]. Around the world, approximately 700 people a day are diagnosed with diabetes, the equivalent of one person every two minutes; approximately 90% of these have type 2 diabetes.

Type 2 diabetes poses a significant public health and healthcare challenge, resulting in the premature deaths of 22,000 people every year. It is a leading cause of preventable sight loss in people of working age and is a major contributor to kidney failure, nerve damage, heart attack and stroke. The cost of diabetes to the National Health Service (NHS) is over £1.5 million an hour, or 10% of the NHS budget for England and Wales. In total, an estimated £14 billion is spent every year treating diabetes and its complications, with the cost of treating complications representing a much higher cost [2].

Evidence suggests that the prevention of type 2 diabetes is achievable through moderate weight loss and lifestyle changes [3], with one kilogram of weight loss being associated with a 16% reduction in the incidence of diabetes [4]. National Institute for Care and Excellence (NICE) guidelines recommend early detection of type 2 diabetes, as well as provision of an evidence-based intensive lifestyle-change programme to prevent or delay the onset of type 2 diabetes for those at high risk [5,6,7,8].

Intensive lifestyle-change programmes offer ongoing tailored advice, support and encouragement to enable people to increase their physical activity, improve diet, and gradually lose weight to reach and maintain a healthy Body Mass Index (BMI). Such changes can, in turn, reduce the risk of developing other long-term conditions. For example, many randomized controlled trials have shown significant modifications in key risk factors for cardiovascular disease and certain cancers as a result of relatively modest reductions in weight (2–3 kg) or an increase in physical activity levels (30–60 min per week of moderate intensity) [9,10,11,12,13].

In 2014, the NHS published its “Five Year Forward View”, stating that a major upgrade in prevention and public health was needed in order to sustain the NHS and the economic prosperity of Britain. As part of this prevention strategy, the NHS Diabetes Prevention Programme (NHS DPP) commenced rollout in England in 2016, with the aim of becoming available to the whole country by 2020. The programme, which is a joint initiative between NHS England, Public Health England (PHE) and Diabetes UK, aims to significantly reduce the 4 million people in England otherwise expected to have type 2 diabetes by 2025. Eligible patients receive evidence-based and personalized help to reduce their risk of type 2 diabetes, including healthy lifestyle education, bespoke physical activity programmes, and help to lose weight (if appropriate).

This article builds upon on steps taken by South Gloucestershire Local Authority to pilot a diabetes prevention programme prior to the rollout of the NHS DPP in the area. It reports on the evaluation of patient outcomes (qualitative and quantitative) immediately at the end of the pilot programme and again at six months. It also aims to report on the process of managing and delivering the programme, to consider lessons learned from the pilot project and how these lessons could be incorporated to ensure the ongoing success of a diabetes prevention programme in the locality. Learning from the pilot is intended not only to help shape the future of diabetes prevention care in South Gloucestershire, but also to have implications for service development in this field.

The local Joint Prevention Strategy and the Long-Term Conditions Strategy have emphasized that obesity, pre-diabetes and type 2 diabetes are priority areas for commissioners of clinical healthcare services. In 2014, South Gloucestershire was estimated to have a population of 271,600 people, with 25 General Practitioner (GP) practices. This presents a substantial challenge in terms of numbers of patients at risk of developing type 2 diabetes. GP practices data across South Gloucestershire highlighted a 6.29% rise in diabetes prevalence in the area between 2014–2015 and 2015–2016 (from 10,954 to 11,690 patients). In 2015–2016, 8.5% (511) patients in South Gloucestershire had a raised glycated haemoglobin (HbA1c) identified during a routine health check.

Historically, diabetes education has been available in the area for those patients newly diagnosed with the condition, but not for patients who are at ‘high risk’ of developing it. Recognizing a gap within the service, and the rising prevalence of type 2 diabetes locally, an application was made to Health Education South West (HESW) for funding to run a one-year pilot Diabetes Prevention Project, from October 2015 to October 2016.

The aim of the pilot project was to identify a group of patients at risk of developing type 2 diabetes within one GP practice and deliver a recognized diabetes prevention programme. The project also aimed to increase staff capacity and knowledge, enabling staff to deliver diabetes prevention education to patients in preparation for when the NHS DPP commenced in the locality.

The South Gloucestershire Diabetes Prevention (Pilot) Programme (SGDPP) was based on X-PERT X-POD (Prevention of Diabetes), an established, manual-based programme comprising a six-week group education course followed by telephone and email support, with meetings scheduled at three months post-course and at six months post-enrolment. The dietary approach recommended by the X-POD programme is low-fat, low-carb, Mediterranean, intermittent fasting, and 500 kcal deficient [14]. The design of the programme is compliant with NICE [6] guidelines on the prevention of diabetes.

## 2. Materials and Methods

Two GP surgeries were chosen for the pilot. The surgeries were selected on the basis of their capacity to offer staff and room facilities for the pilot. Following group consultation, the following criteria for invitation to the programme were agreed: patients must be over 35 years old, and have a previously recorded BMI of over 30 plus fit into one of the following categories: (1) family history of diabetes; (2) previous raised HbA1c blood result (42–47 mmol inclusive); (3) history of cardiovascular disease; (4) hypertension; (5) history of gestational diabetes or polycystic ovary syndrome; or (6) black or minority ethnic group. These criteria were applied to the patient record at both surgeries by practice staff, and 500 patients were identified. Of these, the first 300 listed were sent letters inviting them to the programme. Mail was generated and sent from within the surgery in order not to breach patient confidentiality. Letters explained to patients how to enroll in the course if they would like to do so, with a range of times on offer.

The evaluation was structured around three areas: (1) an outcomes assessment for a sample of participants, (2) a process evaluation of the perceptions of project implementation by participants, project staff and wider stakeholders, and (3) a unit cost analysis.

### 2.1. Outcome Evaluation

This component of the evaluation focused on changes that reflect anticipated outcomes for participants, including objectively-measured changes in weight, waist circumference and HbA1c, and self-reported outcomes for general health, physical activity, dietary behavior, mental wellbeing, mental ill health and social wellbeing. Study participants were all patients who met the eligibility criteria for the diabetes prevention project. A target of at least 30 completed matched pairs of before and after data was set.

Participants completed a baseline questionnaire at enrolment and a follow-up questionnaire at six months. All participants were asked to provide information on their demographics, co-morbidities, and any concurrent participation in other healthy lifestyle activities. These data were analyzed using SPSS Statistics Version 22.0 (IBM, Armonk, NY, USA).

### 2.2. Process Evaluation

The process evaluation examined key aspects of the project implementation, drawing upon theory-driven evaluation methodologies [15,16]. All participants were approached to take part in focus groups or one-to-one interviews. An interview indicative topic guide was piloted and then used for data collection. Audio-recorded data were transcribed and thematically analyzed using NVivo 10 (NVivo, QSR International, Melbourne, Australia). All members of the evaluation team coded and checked the data for consistency by comparing and discussing preliminary findings.

A similar process was used to collect data from project stakeholders through interviews. Project management meetings, the expert panel meeting, and interviews with the GP practice management team were audio-recorded.

### 2.3. Unit Cost Analysis

Methodology used by Savas and Grady to evaluate an Impaired Glucose Regulation (IGR) project in Manchester [17] was adopted to identify a range of costs linked to the SGDPP, including costs of recruiting and enrolling participants, delivering group-based education sessions, providing follow-on support, staff training and development, meetings, programme management and administration, and other worker time.

### 2.4. Ethical Considerations

Prospective participants in the study were provided with written information, given an opportunity to ask questions, and asked to provide written consent. This study obtained ethical approval through South Gloucestershire Council’s Research Governance Framework and through the University of the West of England Research Ethics Committee (Ref: HAS/15/12/066).

## 3. Results

### 3.1. Study Sample

**Figure 1:**
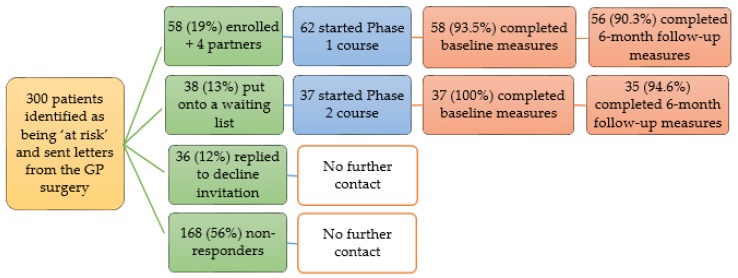
Overview of the SGDPP participant recruitment and retention.

**Figure 2:**
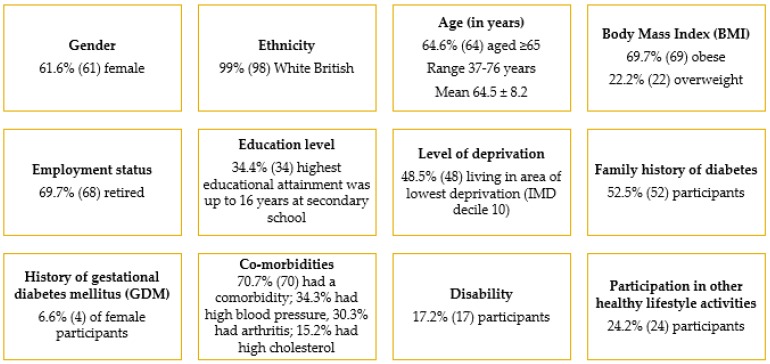
Key characteristics of the 99 participants who started the course.

Three hundred eligible patients were invited onto the programme by letter. Patients responded quickly to express interest in the groups, and initial group places filled rapidly. Some patients were uncertain why they had been chosen, some objected to receiving a letter, while others welcomed the opportunity to learn more about their risk. As demand exceeded expectation, further groups were organized. In the end, a total of 95 (32%) patients enrolled, and 87 (29%) completed the course to the six-month stage. Four of the attending partners discovered they also met the inclusion criteria, and so enrolled and completed the course to the six-month stage. Figure 1 gives an overview of participant recruitment and retention, while Figure 2 shows the key characteristics of the 99 participants who started the course.

Drop-out from the programme and evaluation was very low; only 8% of participants who started the programme were unable to attend and provide data at the six-month follow-up meeting. All group sessions and the six-month follow-up meeting had an attendance of at least 84%, with a 90% mean attendance at group sessions alone. Over sixty three percent (63.6%) of participants attended all six group sessions. Mean contact time at group sessions was 10.7 ± 2.4 h. Overall feedback on the group sessions, provided by participants was very positive—96% scored a 9 or 10 when asked how likely they were to recommend the course to friends or family (where ‘0’ is extremely unlikely and ‘10’ is extremely likely); this is supported by the process evaluation findings presented in Section 3.3.

### 3.2. Outcomes at Six Months

Baseline and six-month follow-up data were available for 91 participants, far exceeding the original target of 30. Table 1 shows that there were significant improvements across all measures, except those relating to mental wellbeing and mental ill health (anxiety and depression). Almost 76% (69) participants experienced weight loss, while 87.9% (80) had reduced HbA1c.

Further analysis found that:

- Males lost more weight than females:Males:
  Males: M = −5.85 kg ± 4.79 kg; Females: M = −2.97 kg ± 4.19 kg; t (89) = −3.00, *p* = 0.003.
- Participants living in more deprived areas lost more weight than those in less deprived areas:
  Higher Index of Multiple Deprivation (IMD) decile: M = −5.58 kg ± 5.45 kg;
  Lower IMD decile: M = −2.95 kg ± 3.58 kg; t (59.3) = −2.60, *p* = 0.012.

Associations between weight loss and age category, employment status and educational level were either absent or could not be determined due to small sample sizes. Additional investigations on weight change at six months were done for the 86 participants who were overweight or obese (BMI ≥ 25 kg/m^2^):

- 60.5% (52) achieved 2 kg weight loss, pre-set as the target for a minimum clinically important difference (MCID).
- Weight loss thresholds widely adopted in weight management programmes are at least 3%, 5% or 10%—the proportion of participants who achieved these thresholds were 52.3% (45), 40.7% (35) and 12.8% (11), respectively.

Results from stepwise multiple regression found that gender, followed by area deprivation, were predictors of weight change at six months (*p* < 0.01). Three other factors—contact time at group sessions, comorbidities, and prior and/or concurrent engagement with other healthy lifestyle activities—were not predictors of weight change.

### 3.3. Process Evaluation Findings: Participants

Data were collected from 67 participants through 14 focus groups, five one-to-one interviews and one written feedback. They suggest that aspects of the project that were vital to its success were organization, clarity of goals and personal qualities of the delivery team. Positive elements of participant feedback are summarized in Table 2. A majority of participants completed the programme and attended focus groups. This suggested that the intervention was well-received.

#### Suggestions for Programme Development and Rollout

Participants recommended that project staff be ready to respond to requests for more information, following mail-outs of the initial letter of invitation. This was seen as a crucial moment for patient engagement to address any feelings of surprise or shock. The programme itself would benefit from revisions to make some aspects of the education more comprehensible, particularly the pathophysiology of diabetes and dietary advice. However, participants recognized that this was challenging given the nature of the subject matter. Some participants suggested more emphasis on goals and strategies for increasing physical activity levels. Opportunities for inter-group networking and for maintaining contact after the programme were recommended to promote sustainability of behavior change and knowledge sharing as part of wider community initiatives.

### 3.4. Process Evaluation Findings: Staff and Wider Stakeholders

Three delivery team members, two trained patient representatives and two GP surgery staff participated in interviews and provided written feedback. An expert panel of ten practitioners (external to the programme) representing local authority, GP practices, local Clinical Commissioning Groups, and community and voluntary sector agencies, provided feedback on preliminary evaluation.

Staff and wider stakeholders perceived that the pilot project performed very strongly. Take-up (at 29%) and retention (at 92%) were considerably higher than the steering group anticipated; based on work in other areas, the group anticipated recruiting no more than 20% of invitees and retaining under 80% of course participants at six months. However, the GP surgery catchment areas do not include areas of high relative deprivation in contrast to many GP practices in the local region. Staff and stakeholders felt recruitment and retention would be more challenging if the project had been delivered in areas of higher deprivation.

The delivery team had no prior experience of running a diabetes prevention project. However, they had an extensive track record in running similar lifestyle education initiatives to draw upon and combined this with expert input from partners, such as the Diabetic Specialist Nursing Service. The project benefited from a well-briefed, engaged and supportive GP practice team that had an essential role in identifying eligible patients and managing the initial mail-out. Practice staff did not perceive any undue burdens on work resulting from the project. Considerable effort was put in by the delivery team to build relationships with participants both during and after the group stage of the project. Staff appreciated the trailblazing nature of the pilot for the locality and recognized the potential benefits of involving participants in the programme’s development.

### 3.5. Unit Cost Analysis

The NHS DPP intervention costs are estimated to be £270 per person over an 18-month period; these are weighted in the first six months and exclude expenditure on case-finding and referrals to the programme [18]. For the SGDPP, the estimated cost per person was £167, including project set-up, delivery, administration and management over six months. Sensitivity analysis found the cost per person ranged from £107 to £495, with higher costs including X-PERT training, equipment and evaluation.

## 4. Discussion

This evaluation of the SGDPP has identified some highly promising aspects of the pilot programme. Outcome data show that the programme was associated with positive changes in weight, HbA1c, diet and physical activity measures across a wide distribution of demographic characteristics. The mean weight loss of 4.3% at six months was comparable to the 4% weight loss reported from the systematic review and meta-analysis of twenty-eight US diabetes prevention translation studies, albeit the latter was achieved at 12 months [19]. Given that the landmark US Diabetes Prevention Program trial established weight loss as the single most important factor in reducing diabetes incidence (16% reduction in diabetes incidence for every kilogram of weight loss [20]), the 4.04 kg weight loss achieved in the community-based SGDPP is a key result.

The SGDPP exceeded its initial targets for recruitment, delivery and retention. Clear goals, high quality organization, and personal qualities of the delivery team were identified as important features supporting the strong programme uptake and participant retention, satisfaction with the group elements, and participants’ commitment to achieving goals that directly reduce their risk of diabetes. Feedback and patterns of retention suggest that the programme was generally very well-received, not only by participants but by the GP surgery team and associated health and community services. Staff at the main participating GP surgery anticipated that the initiative could be readily adopted in similar settings provided there were similar resources, project staff skills and preparatory activities with the surgery.

There is some evidence supporting the efficiency and cost-effectiveness of diabetes education in a group setting compared with an individual setting [21]. The NHS DPP programme guidance emphasizes that the retention and adherence to NICE programme delivery guidance are critical factors in achieving cost-effectiveness. The start-up unit costs of the SGDPP appear to be similar to other pilot intensive preventative lifestyle programmes and are anticipated to reduce over time as delivery becomes embedded at the local level.

The SGDPP and NHS DPP follow a screen and treat approach, in which a subpopulation is identified as ‘high-risk’ and offered individual intervention. This is one of the two approaches to preventing type 2 diabetes, the other being the public health approach of changing behavior in a whole population at risk through policies on environmental moderators (sociocultural influences, socioeconomic influences, transport, green spaces) [22]. It is important to understand and evaluate complex interventions such as the SGDPP, and then be able to systematically embed evaluation findings into routine practice to improve the quality and effectiveness of health services and care [23]. Nevertheless, research suggests that the screen and treat approach needs to be complemented by population-wide public health approaches to have substantial impact on the worsening of type 2 diabetes [22].

### 4.1. SGDPP Areas for Development

Participants and stakeholders identified several areas of the programme that require further development. Firstly, there is the challenge of anticipating and appropriately addressing any negative reactions or queries to the initial GP letter of invitation. Contents of the programme may need to be revised to reduce the complexity of the diabetes pathophysiology education and dietary advice, and to have more emphasis on physical activity and behavior change techniques. The programme could have included greater focus on smoking cessation, blood pressure and lipid management. There was scope to refine the role of the expert patient and any co-facilitators, especially with regards to physical activity. It is also important to consider the longer-term development of the programme in terms of networking, support and community-based activities. Since the setting for the pilot was not an area of high social deprivation or high racial-ethnic diversity, the programme may need further adaptation or revision if delivered in areas with a different demographic profile.

### 4.2. Strengths and Limitations of the Evaluation

The SGDPP evaluation was done on a small scale, and only tracked participants’ progress up to six months and overall programme delivery over 12 months. Due to limited resources for the evaluation, multimode approaches or follow-up phone calls could not be implemented alongside the invitation letters to increase the response rate. Limited resources also meant that a before-and-after study design was employed, therefore precluding a comparison between participants’ outcomes and normal care. However, this study design allowed for tests of association between baseline and follow-up characteristics and enabled the role of demographic and programme-linked variables in predicting retention to be assessed. The evaluation might have included longer-term follow-up measurements, particularly of weight and HbA1c. The process evaluation component was essential in helping to develop an account of how and why the programme may be contributing towards its stated goals and of the context within which the programme was implemented. Nevertheless, it is important to be aware that the study provides limited insight into the outcomes of the programme if it was to be scaled-up and replicated in other settings.

## 5. Conclusions

The success of pilot projects such as the SGDPP and its evaluation rely on close partnerships with all stakeholders, including local authority public health, clinical commissioners, primary care and the voluntary sector. The SGDPP evaluation provides evidence of reduced risk markers for type 2 diabetes, positive impacts for dietary and physical activity, and potential cost-effectiveness for this format of group-based diabetes prevention intervention. Recruitment, retention and six-month outcomes surpassed initial expectations. Feedback from participants, staff and stakeholders was positive, and has provided insight on how to successfully embed and scale-up the delivery of diabetes prevention work. This evidence enables the integration of learning into a local service delivery and provides a basis to support development and scale-up of the NHS DPP.

## Figures and Tables

**Table 1:** Changes in means from baseline to six-month follow-up for main outcome measures.

Outcome Measure	Baseline Mean (SD)	6-Month Mean (SD)	Mean Difference	Std. Deviation Difference	95% CI of Difference
Weight (kg)	93.50 (16.38)	89.45 (16.28)	−4.04 **^,2^	4.62	−5.01, −3.08
BMI (kg/m^2^)	33.81 (5.95)	32.38 (6.10)	−1.43 **	1.62	−1.77, −1.09
Waist circumference (cm)	107.37 (12.22)	102.05 (12.50)	−5.32 **	5.03	−6.37, −4.27
HbA1c (mmol/mol)	42.24 (5.26)	38.79 (3.46)	−3.45 **	4.07	−4.30, −2.60
Proportion meeting recommended physical activity guidelines ^1^	0.16 (0.37)	0.30 (0.46)	0.13 *	0.48	0.03, 0.23
Mean no. of min/week of MVPA	189.43 (275.26)	421.66 (498.15)	232.23 **	488.15	130.57, 333.89
Overall self-reported dietary behavior	49.68 (7.38)	47.24 (7.46)	−2.45 **	5.93	−3.68, −1.21
Mental wellbeing (max score = 35) ^3^	27.59 (5.04)	27.64 (5.02)	0.04	3.61	−0.71, 0.80
Anxiety (max score = 28)	10.00 (3.91)	9.74 (3.41)	−0.26	2.89	−0.86, 0.34
Depression (max score = 36)	12.87 (4.93)	12.49 (4.42)	−0.37	3.19	−1.04, 0.29

* *p* < 0.01; ** *p* ≤ 0.001. ^1^ At least 150 min/week of moderate-to-vigorous physical activity (MVPA). ^2^ Equivalent to 4.3% mean weight loss. ^3^ max = maximum.

**Table 2:** Summary of positive elements of qualitative participant feedback in focus groups.

General Aspects of the Programme	Experience of Sessions and Educators	Impact on Individual	Other Positive Elements
Overall clarity of key messages Clear format and organization of the programme	‘Soft’ and ‘hard’ skills of group educators Personalized support on specific matters ‘Professional’ handling of sensitive issues Peer support and opportunities for participation	Personal relevance and importance of the programme Perceived influence of the programme on knowledge, skills, attitudes, motivations to change and confidence to manage risk Shared learning beyond the group: family, friends and acquaintances	Diabetes prevention is a very important issue to disseminate locally, not just with at-risk groups, but with the wider public (including children) and professionals Some participants felt motivated to help professionals in this work
